# Berry Leaves: An Alternative Source of Bioactive Natural Products of Nutritional and Medicinal Value^†^

**DOI:** 10.3390/antiox5020017

**Published:** 2016-06-01

**Authors:** Anastasia-Varvara Ferlemi, Fotini N. Lamari

**Affiliations:** Laboratory of Pharmacognosy and Chemistry of Natural Products, Department of Pharmacy, University of Patras, Patras 26504, Greece; abferlemi@upatras.gr

**Keywords:** Vaccinium, Ribes, Rubus, traditional use, polyphenols, chlorogenic acid, analysis

## Abstract

Berry fruits are recognized, worldwide, as “superfoods” due to the high content of bioactive natural products and the health benefits deriving from their consumption. Berry leaves are byproducts of berry cultivation; their traditional therapeutic use against several diseases, such as the common cold, inflammation, diabetes, and ocular dysfunction, has been almost forgotten nowadays. Nevertheless, the scientific interest regarding the leaf composition and beneficial properties grows, documenting that berry leaves may be considered an alternative source of bioactives. The main bioactive compounds in berry leaves are similar as in berry fruits, *i.e.*, phenolic acids and esters, flavonols, anthocyanins, and procyanidins. The leaves are one of the richest sources of chlorogenic acid. In various studies, these secondary metabolites have demonstrated antioxidant, anti-inflammatory, cardioprotective, and neuroprotective properties. This review focuses on the phytochemical composition of the leaves of the commonest berry species, *i.e.*, blackcurrant, blackberry, raspberry, bilberry, blueberry, cranberry, and lingonberry leaves, and presents their traditional medicinal uses and their biological activities *in vitro* and *in vivo*.

## 1. Introduction

The everlasting quest for health promoting and disease preventing agents in the developed world has changed our view of food sources; superfoods, functional foods, food supplements, and nutraceuticals were introduced and have enriched the products of the food industry contributing to its further growth [1]. Berry fruits constitute a large group of functional food or “superfoods”, whose consumption delivers several health benefits beyond basic nutrition, but little is known about the leaves of berry plants. In this review, we present the phytochemical composition of the leaves of common berry species, as well as summarize their traditional medicinal uses and the results of the evaluation of their biologic properties *in vitro* and *in vivo* so far.

In order to compile the review, a search was performed in the PubMed (http://www.ncbi.nlm.nih.gov/pubmed) and Google Scholar databases. The search terms included the keywords: berries, Vaccinium; Ribes; Rubus, and the common names of each species (the headings of all chapters of this review) and leaves. In January 2016, this search yielded about 500 results in the PubMed database. Information on the traditional uses has also been acquired from the European Medicines Agency (EMA) monographs on the respective herbal medicines (http://www.ema.europa.eu). Original research and review articles were taken into account and special emphasis was placed on published literature concerning the few available clinical studies.

The small edible, brightly colored berries are low energy density fruits, rich in vitamins, fibers, and various phenolic compounds [2]. The edible berries belong to the genera of *Vaccinium* (blueberries, cranberries, bilberries, lingonberries), *Ribes* (gooseberries, black and red currants), *Rubus* (raspberries, blackberries and cloudberries), *Fragaria* (strawberries), *Aronia* (chokeberries), and *Sambucus* (elderberries). All of them contain high levels of phenolics, which greatly contribute to their organoleptic properties and health benefits.

**Figure 1:**
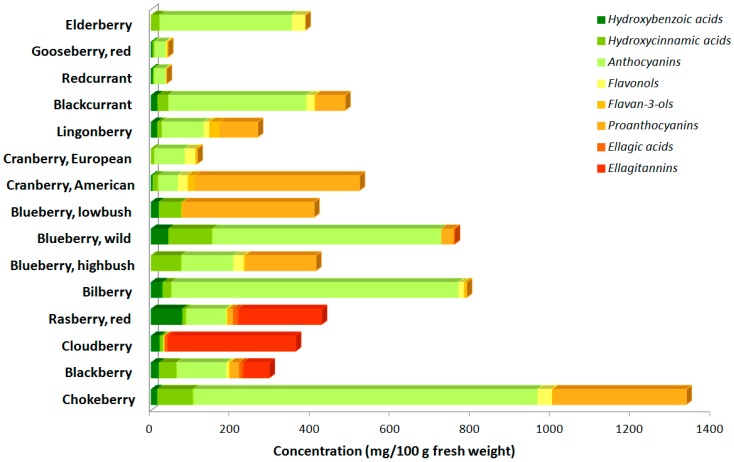
The phenolic composition of the commonest berries. Chokeberries (*Aronia mitschurinii)* contain the highest concentration of bioactive compounds, mainly anthocyanins and proanthocyanins. Bilberry (*Vaccinium myrtillus*) is rich in anthocyanins, as well as wild blueberry (*Vaccinium corymbosum*), which also contains notable amount of phenolic acids. Lowbush blueberry (*Vaccinium angustifolium*) and the American cranberry (*Vaccinium macrocarpon*) are sources of proanthocyanidins. The berries of the genus *Rubus*, *i.e.*, raspberry (*Rubus idaeus*), cloudberry (*Rubus chamaemorus*), and blackberry (*Rubus fruticosus*) contain all the principle bioactive compounds that we meet in berries, and especially ellagitannins. Gooseberry (*Ribes uva-crispa*) and red currant (*Rubus rubrum*), on the other hand, contain mainly phenolic acids and only traces of the other compounds.

Berry phenolics represent a diverse group of compounds including phenolic acids (hydroxybenzoic and hydroxycinnamic acids, and their derivatives), flavonoids, such as flavonols, flavanols, and anthocyanins, and tannins (gallotannins and ellagitannins), divided into condensed tannins (proanthocyanidins) and hydrolysable tannins. A greater variety of compounds is recorded for blackberries and raspberries of the genus *Rubus*, whereas all of the other berry species are usually characterized by the high levels of specific phenolic groups, *i.e.*, anthocyanins and proanthocyanidins (Figure 1). The berries of genus *Rubus*, as well as the chokeberries, are often richer than other berries in *p-*hydroxybenzoic acids; they also contain moderate levels of hydroxycinnamic acids; however, trace amounts were detected in cloudberries [3,4,5,6]. In contrast, blueberries (*Vaccinium* spp.) are the richest source of hydroxycinnamic acids, such as *p-*coumaric acid, chlorogenic acid, and other caffeic acid derivatives [3,4,6,7,8]. With respect to flavonoids, chokeberries, highbush blueberries, American cranberries (*V. macrocarpon*), blackcurrants, and lingonberries contain the highest concentration of flavonols, especially quercetin and myricetin derivatives and aglycones [3,5,6,7,8]. On the contrary, raspberries, cloudberries, red currants, and gooseberries contain only traces of flavonols [4,5].

The bright color of the small edible berries is attributed to the significant quantities of anthocyanins, which are distributed mainly to the epidermal tissues in fruits. Substantial amounts of different types of anthocyanins (glycosylated or not) are found in chokeberries, bilberries, wild and cultivated blueberries, elderberries, blackcurrants, and the European cranberries (*V. oxycoccus*). Bilberries, for example, contain fifteen different anthocyanins, *i.e.*, delphinidin and cyanidin monoglycosides (the principal anthocyanin), petunidin, peonidin, and malvidin glycosides. Strawberries contain mainly pelargonidins, while lingonberry, red currant, chokeberry and elderberry anthocyanins consist only of cyanidin glycosides. Cyanidins are also the principal anthocyanins in American cranberries, while in European cranberries the most abundant are peonidins [4,5,6,8,9,10].

Proanthocyanidins, consisting only of procyanidins, *i.e.*, (+)-catechin and (−)-epicatechin polymers, are present in high concentration in chokeberries, high- and lowbush blueberries, American cranberries, and lingonberries [4,11]. Finally, ellagic acids and ellagitannins are in high amounts in the berries belonging to the genus *Rubus* (cloudberries, raspberries), as well as in strawberries [5,10]. Figure 1 summarizes the phenolic content of the most common berry fruits.

A strong body of scientific research documents the contribution of the consumption of berries to the three targets of functional foods: (i) health maintenance (e.g., mental health, immune function); (ii) reduced risk of obesity; and (iii) reduced risk of chronic diet-related diseases (e.g., cardiovascular disease, type 2 diabetes, and metabolic syndrome) [1]. However, not only the fruits, but also the leaves, of the berry plants have been used in traditional remedies; leaf extracts have often been used against several diseases, such as colds, inflammation of the urinary tract, diabetes, and ocular dysfunction by Native Americans and other populations, but these treatments have been almost forgotten nowadays. In the last five years, the European Medicines Agency (EMA) has approved the circulation of leaf infusions and extracts of *Ribes nigrum*, *Rubus idaea*, and *Arctostaphylos uva-ursi* as herbal medicinal products based on their traditional uses and another monograph for the wild strawberry (*Fragaria vesca* L.) leaf extracts has just been announced [12,13,14,15].

**Figure 2:**
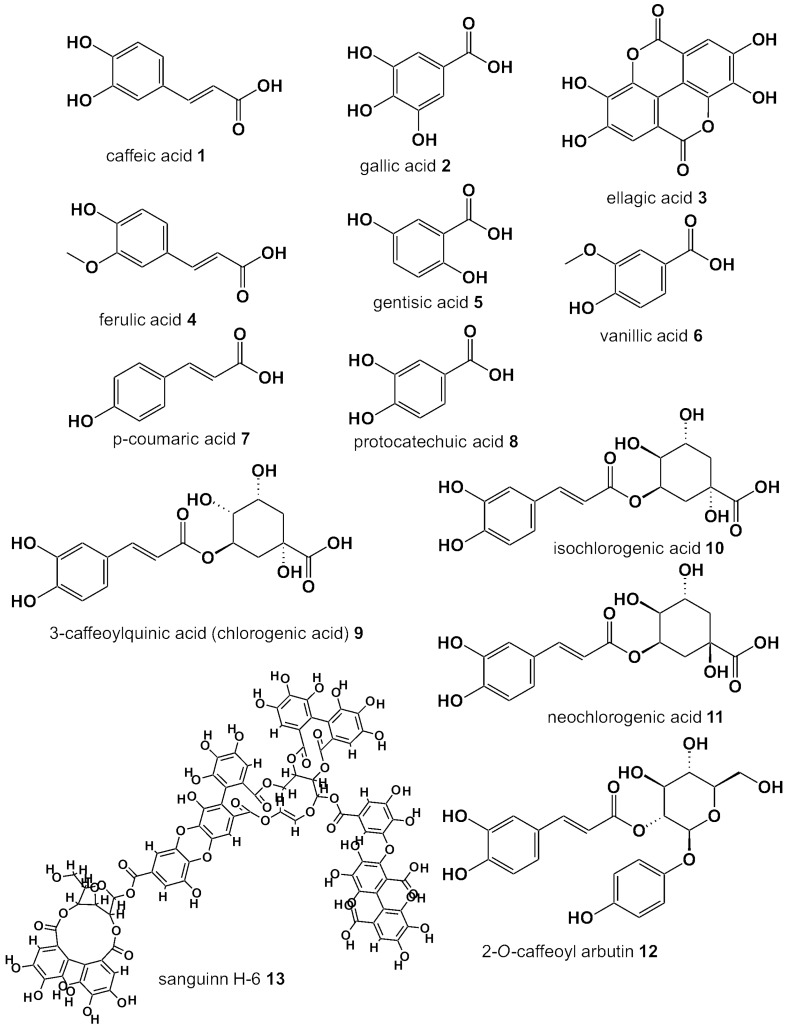
Structures of the most common phenolic acids and acid derivatives of berry leaves.

**Figure 3:**
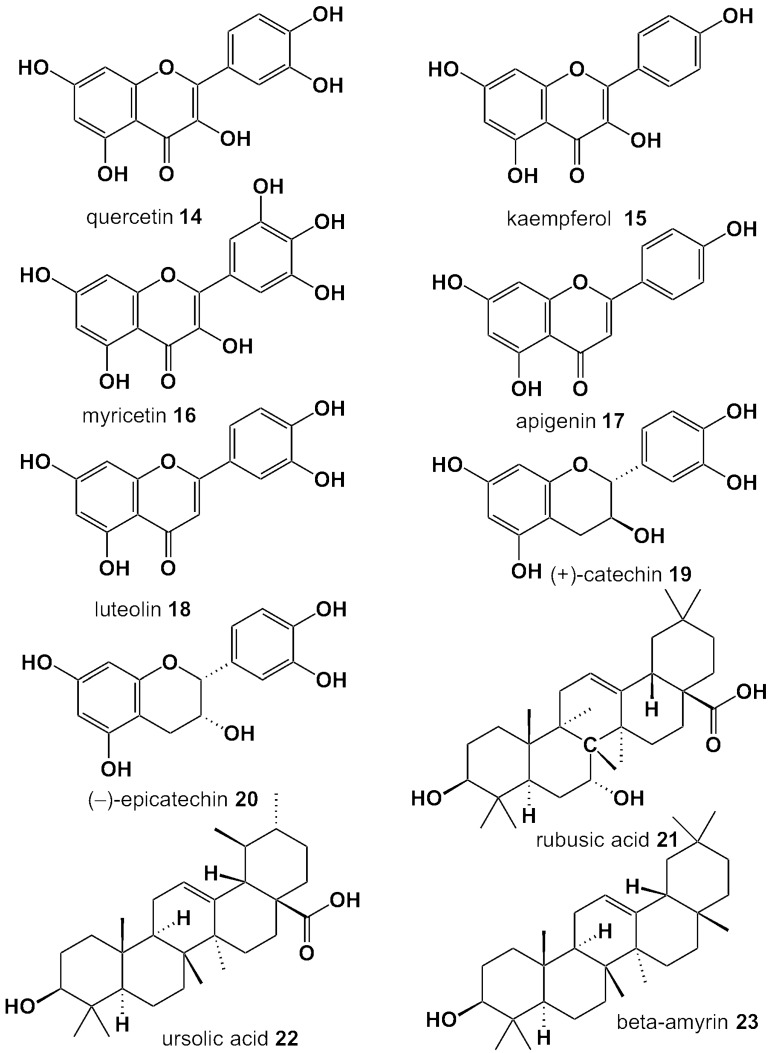
Structures of the main flavonoid aglycons and terpenes of berry leaves.

Despite their medicinal value, which stems in large from their phenolic/polyphenolic content, berry leaves are the main byproducts of harvesting, meaning that tons of leaves are wasted annually. Analytical studies show that the leaf phenolic composition is similar to that of the precious fruits or even richer and higher, indicating that they may be utilized as an alternative source of bioactive natural products for the development of food supplements, nutraceuticals, or functional foods. This review presents our knowledge heretofore; we present the phytochemical composition of the leaves of the common berry species, as well as summarize the studies of the beneficial activities of their extracts pertaining to their nutritional or medicinal value. The compositions of the berry leaves are summarized in Table 1. The structures of the commonest phenolic acids and derivatives are presented in Figure 2 and of the flavonoid aglycons and terpenes in Figure 3. The traditional medicinal uses and the relevant biological properties demonstrated by *in vitro*, *in vivo*, and clinical studies are presented in each subsection, but also in tabulated form (Table 2).

## 2. Blackcurrant (*Ribes nigrum*) Leaves

In 1996, French Pharmacopoeia included, for the first time, a monograph on *Ribes nigrum* L. folia [48] and in 2010, EMA issued the official community monograph for blackcurrant leaves [14].

The slightly wrinkled leaf is dark green at the upper surface and pale greyish green at the lower surface, on which a widely spaced reticulate venation is particularly distinct. Moreover, the leaves have glands that can be seen as scattered yellowish dots. In contrast with the fresh leaves that are strongly aromatic, the dried leaves have no odor or taste. The leaves should be collected during or shortly after flowering [48].

The phytochemical analysis based on spectroscopic and separation techniques revealed that the most abundant secondary metabolites of blackberry leaf can be subdivided into three groups: hydroxycinnamic acids, flavonoids, and proanthocyanidins. In detail, experiments with thin layer chromatography (TLC) by Trajkovski in 1974 revealed that chlorogenic acid (9) and its isomers (iso- and neo-chlorogenic acid, 10 and 11) are found in relatively high amounts in the foliage of blackcurrant [49]. Other hydroxycinnamic acids in these leaves are caffeic acid (1), gallic acid (2), ferulic acid (4), coumaric acid (7), and gentisic acid (5) [49]. In a recent HPLC-DAD analysis of the ethanolic extract of blackcurrant leaves, only chlorogenic acid and its isomer neo-chlorogenic acid were quantified; their concentrations ranged from 0.081 to 0.121 mg/g dry weight and from 0.044 to 0.435 mg/g dry weight, respectively [50]. In an ethanolic extract of blackcurrant leaves from Estonia, chlorogenic acid concentration was found much higher (14.93 mg/g dried leaves) by HPLC-MS analysis [20]. With regards to flavonols, kaempferol (15) and quercetin (14) derivatives have been reported in the foliage of *Ribes nigrum* [49]. Particularly, Vagiri *et al.* [50] have identified quercetin-3-*O*-rutinoside (0.099–0.229 mg/g dry weight), quercetin-3-*O*-galactoside (0.057–0.081 mg/g dry weight), quercetin-3-*O*-glucoside (0.038–0.085 mg/g dry weight), and the most abundant flavonoid, quercetin-3-*O*-malonylglucoside (2.424–3.890 mg/g dry weight). Additionally, kaempferol-3-*O*-rutinoside (0.019–0.036 mg/g dry weight), kaempferol-3-*O*-glucoside (0.017–0.031 mg/g dry weight), kaempferol-3-*O*-malonylglucoside (0.135–0.409 mg/g dry weight), an isomer of the latter (0.488–2.441 mg/g dry weight), as well as myricetin-malonylglucoside (0.042–0.055 mg/g dry weight) and its isomer (0.019–0.023 mg/g dry weight) were quantified [50]. The range of values depends on the harvest time [50]. The HPLC-MS analysis of the Estonian blackcurrant leaf extract showed that quercetin glucoside (quercetrin) and rutinoside (rutin), the two major glycosides of the ethanolic extract, were found in high concentrations (19.47 and 3.99 mg/g dried leaves, respectively) [20]. Furthermore, isorhamnetin-3-*O*-rutinoside, isorhamnetin-3-*O*-glucoside, kaempferol-3-*O*-galactoside and kaempferol-3-*O*-glucosyl-6’’-acetate have been recorded in the ethanolic extract of *Ribes nigrum* leaves [19,51]. Catechin (19), epigallocatechin, and epicatechin (20) have also been detected in the leaves even though not constantly; the concentration of catechin in the ethanolic extract of Estonian leaves was 7.89 mg/g [20]. In a methanolic extract of blackcurrant leaves, Tits *et al.* [52,53] have identified, via high performance thin layer chromatography (HPTLC), nuclear magnetic resolution (NMR), and infrared spectroscopy (IR), several different tannins (catechin, epicatechin, gallocatechin, epigallocatechin, gallocatechin-(4a-8)-gallocatechin, gallocatechin-(4a-8)-epigallocatechin, and the new trimer: gallocatechin-(4a-8)-gallocatechin-(4a-8)-gallocatechin, gallocatechin-(4a-8)-catechin, gallocatechin-(4-6)-gallocatechin, and catechin-(4a-8)–gallocatechin-(4a-8)-gallocatechin).

A significant difference between fruits and leaves is the lowest concentration of anthocyanins in the latter; delphinidin-3-*O*-glucoside and –rutinoside, as well as cyanidin-3-*O*-glucoside and –rutinoside have been identified, but not quantified, in the ethanolic extract of blackcurrant leaves [51]. Another difference is the absence of hydroxybenzoic acids. However, the blackcurrant leaves contain significant substances, such as glycerolipids (mainly alpha-linoleic acid) and ascorbic acid (1–2.70 mg/g dried material) [54]. Finally, the essential oil of the leaves of *Ribes nigrum* contains mainly monoterpenic substances (α-pinene, myrcene, *p*-cymene, limonene, β-ocimene, *etc.*), the sesquiterpenes caryophylene and humulene, as well as methyl salicylate [55].

According to the EMA monograph on *Ribes nigrum* folium, its tea is a traditional medicinal product for minor articular pain and a diuretic to achieve flushing of the urinary tract as an adjuvant in minor urinary complaints [14]. The leaves of blackcurrant have been used in folk medicine for their diaphoretic properties, as well as against diarrhea and spasmodic cough [20,54]. The leaves have significant antioxidant and anti-inflammatory properties (inhibition of myeloperoxidase activity and reactive oxygen species production on activated neutrophils), as it has been demonstrated by biologically-relevant cellular models, which substantiate their traditional uses against inflammatory conditions [54,56]. These effects are correlated with the phenolic ingredients that are originally synthesized by plants to protect themselves from pathogens [57]; therefore, it has been proposed that *Ribes nigrum* leaves might be used for large scale extractions of antioxidant molecules [56]. Furthermore, evidence from carrageenan-induced rat paw edema studies has revealed the potential analgesic properties of blackcurrant leaves, which was later reinforced by findings of blackcurrant proanthocyanidin inhibition of leukocyte infiltration [20].

## 3. Blackberry (*Rubus fruticosus*) Leaves

Blackberries are perennial shrubs, lasting for three seasons or more. The upper side of the leaves is dark green, while the underside is lighter green. Short prickles cover the stalks and veins of the leaves [25].

Many phytochemical investigations have proven the presence of diverse secondary metabolites in blackberry leaves. In general, they are rich in tannins and they, also, contain a notable amount of flavonoids, phenolic acids, triterpenes, mineral salts, and vitamin C [22,23,25]. More specifically, phenolic acids like ellagic (3), gallic (2), caffeic (1), and *p*-coumaric (7) acids, flavonoids, such as quercetin (14), hyperoside, kaempferol (15), myricitin, (+)-catechin (19), (−)-epicatechin (20), epicatechin gallate, and proanthocyanidin B1 have been identified in the leaves of *R. fruticosus*, as well as in the fruit [25]. The HPLC analysis of a hydrolyzed methanolic extract of blackberry leaves showed that total flavonoids, expressed as quercetin equivalents, range from 0.14% to 0.31% of dry weight, while total ellagic acid ranges from 2.93% to 4.32% of dry weight [24].

In 2015, Ozmianki *et al.* [22] extensively analyzed the phenolic composition of twenty-six different wild blackberry leaf samples by LC/MS QTOF; 33 compounds were detected in the respective methanolic extracts, *i.e.*, 15 flavonols, 13 hydroxycinnamic acids, three ellagic acid derivatives, and two flavones. The total content of phenolic compounds extracted from the leaves of wild blackberries, calculated as the sum of compounds resulting from UPLC-PDA analysis, was highly diverse and ranged from 83.02 mg/g dry matter for *R. austroslovacus* to 334.24 mg/g dry matter for *R. perrobustus*. The largest group of phenolic compounds was that of ellagitannins (51.59–251.01 mg/g dry matter), as in blackberry fruits [52]; the most abundant ellagitannins in the wild blackberry leaves were sanguiin H-6 (13) (range 0–73.92 mg/g dry matter), lambertianin C (range 16.75–123.41 mg/g dry matter), and casuarinin (34.47–117.86 mg/g dry matter). In the same study, the second group of bioactive compounds in the leaves of wild blackberries was that of derivatives of quercetin (14), kaempferol (15), luteolin (18), and apigenin (17) (average content 35.17 mg/g dry matter); kaempferol-3-*O*-glucuronide and quercetin-3-*O*-glucuronide (9.23 and 7 mg/g dry matter, respectively) were the most abundant compounds. The next group of compounds in blackberry leaves is composed of phenolic acids, especially derivatives of caffeic acid (1), *p*-coumaric acid (7), and ellagic acid (3) (average content 28.74 mg/g dry matter); *p*-coumaric acid derivatives and neo-chlorogenic acid (11) were found in notable amounts in blackberry leaf extracts [22].

Robinson *et al.* [26], in 1931, reported the presence of cyanidin-3-*O*-saccharide in blackberry leaves. Almost 40 years ago, two triperpene acids were isolated from *R. fruticosus* leaves, rubusic (21) and rubinic acids; 2-a-hydroxyursolic acid and β-amyrin (23) were also detected [18,27].

Health-promoting effects and immunity-boosting properties have been attributed to blackberry leaves since long ago. Hippocrates recommended blackberry stems and leaves soaked in white wine for facilitating childbirth [16]. Zia-UI-Haq *et al.* [25], in their review of the traditional uses of *Rubus fruticosus* leaves, reported that the decoction of the leaves has been used as tonic and a mouthwash; gargles help treating thrush, gum inflammation, sore throat, and mouth ulcers. The leaves are also chewed in order to strengthen the gums and to cure thrush. A poultice of the leaves is applied to abscesses and skin ulcers as an astringent. In addition, blackberry leaves and roots are a long-standing home remedy for anemia and menses, diarrhea, dysentery, cystitis, and hemorrhoids. Finally, they have traditionally been used against several respiratory problems [19].

Indeed, it has been demonstrated that the leaves of blackberry possess significant antimicrobial activity, higher than the fruit, against several bacterial strains, such as *Salmonella typhi*, *Escherichia coli*, *Staphylococcus aureus*, *Micrococcus luteus*, *Proteus mirabilis*, *Bacillus subtilis*, *Citrobacteri* sp., and *Pseudomonas aeruginosa* [25]. In contrast, when the methanolic extracts of blackberry leaves were tested for their antifungal potential against nine pathogenic fungal strains (*Yersinia aldovae*, *Aspergillus parasiticus*, *Candida albicans*, *Aspergillus niger*, *Aspergillus effusus*, *Macrophomina phaseolina*, *Fusarium solani*, *Trichophyton rubrum*, *Saccharomyses cerevisiae*) they did not have any biological activity [17].

Several studies point out the anti-diabetic effect of blackberry leaf extracts; water and butanol extracts were reported to be active in non-insulin dependent diabetes and had significant hypoglycemic effect in normal rats [58]. Similar results were obtained for the infusion of blackberry leaves in alloxan-diabetic rabbits [59]. Moreover, a tea made from *R. fruticosus* leaves decreased diabetic symptoms (hyperglycemia), a property partly attributed to their content in chromium and zinc [60]. Finally, the antioxidant and angiogenic activities of different extracts of blackberry leaves have also been recorded in several studies [22,28,29].

## 4. Raspberry (*Rubus idaeus*) Leaves

The green leaves of *Rubus idaeus* have been included in British Pharmacopoeia since 1983 [30] and in 2012 the European Medicines Agency issued a community herbal monograph on red raspberry leaves [13].

The beneficial medicinal properties are attributed to the bioactive compounds of the leaves, which are mainly hydrolysable tannins [21]. Gudej [24] reported that tannin concentration in the dried raspberry leaf ranges from 2.6% to 6.9% (*w*/*w*) and that the principle compounds are ellagic acids. Additional ellagitannins that have been identified in these leaves are the dimers sanguiin H-6 (13) and H-10, and the trimers lambertianin D and lambertianin C, as well as methyl gallate [19,21]. The second most abundant group in raspberry leaves is flavonoids. The quantity of flavonoids in the leaves of *R. idaeus* is significantly higher than that in the fruits where flavonoids compose only a very small fraction of the bioactive compounds; leaf flavonoids range from 0.46% to 1.05% (*w*/*w*) [31]. In the study of Ozmianski *et al.* [19] the flavonoid fraction was the main phenolic group, constituting almost 11% of leaf extract powder weight.

Phenolic acids, other than ellagic acid (3), have been found in very small amounts, mainly caffeic (0.55 mg/g dried leaf) (1) and chlorogenic acid (0.70 mg/g dried leaf) (9) [61]. Moreover, *p*-coumaric (7), ferulic (4), protocatechuic (8), gentisic (5), caffeoyltartaric, feruloyltartaric, and *p*-coumaroyl-glucoside acids, as well as *p*-hydroxybenzoic and vanillic acids have been reported in raspberry leaves [62]. Finally, terpenoids have been identified, including mono- and sesquiterpenes, like terpinolene and 3-oxo-α-ionol, as well as triterpenes, such as a- and β-amyrin (23), squalene and cycloartenol [6,21].

The study of Gudej [24] presents an interesting comparison of the main *Rubus* categories, *i.e.*, blackberries and raspberries. The leaves of wild raspberry (*R. saxatilis*), cultivated raspberry species (*R. idaeus* Malling Promise) and blackberry (*R. fruticosus* Gazda) had the highest flavonoid content as measured by HPLC. Furthermore, the leaves of raspberries are characterized by lower amounts of both tannins and ellagic acid (3.25% and 2.53% of dry weight respectively) than blackberry leaves (6.50% and 4.32% of dry weight respectively) [24].

Raspberry leaf has been used in Europe for various gynecological disorders, *i.e.*, menstruation, labor and ailments of the gastrointestinal tract (diarrhea) [21,30]. It is reported that a hot tea made from raspberry leaves stimulates and facilitates labor and shortens its duration [21,63]. Other traditional uses include its use as an astringent gargle and less often for chronic skin conditions and for the treatment of conjunctivitis [30]. The European monograph on raspberry leaf has approved its use as a traditional herbal medicinal product for the symptomatic relief of minor spasms associated with menstrual periods, for the symptomatic treatment of mild inflammation in the mouth or throat, and of mild diarrhea [13].

Since raspberry leaf is a commonly used herb during pregnancy today, earlier and current investigations have explored its effects mainly regarding labor. Jing *et al.* [64] reported that pretreatment of pregnant rats with tea did not alter the ability of oxytocin to initiate contractions. Additionally, in pregnant animals treated with red raspberry leaf tea, labor was not augmented by a direct effect on uterine contractility; in contrast, it had variable effects on preexisting oxytocin-induced contractions, sometimes augmenting the effect of oxytocin and sometimes causing augmentation followed by inhibition [64]. Furthermore, these effects depended on the herbal preparation used and on pregnancy status [64].

Two different clinical studies were performed in order to assess the efficacy of raspberry leaf preparations in pregnancy [63,65]. About 150 women were included in the studies. No clinically significant differences were observed among the different groups regarding maternal blood loss, maternal diastolic blood pressure pre labor or transfer to special care baby unit, length of gestation, the likelihood of medical facilitating of labor, and need for pain relief during labor. In addition to these studies, others report that raspberry leaves possess significant antioxidant activity, stronger than the respective extracts of blackberry leaves [29].

## 5. Bilberry (*Vaccinium myrtillus*) Leaves

Bilberries are one of the most important wild berries in Northern Europe, commonly called European blueberries to distinguish them from the other blueberries. Qualitative and quantitative analysis studies based on LC/MS conclude that the main bioactive compounds of bilberry leaves are hydroxycinnamic acids (36% of the weight of leaf extract powder) and especially chlorogenic acid (9) [19,34,66]; its concentration ranges from 59% to 74% of the total hydroxycinnamic acids [66]. Jaakola *et al.* [37] and Riihinen *et al.* [35] reported that the concentration of hydroxycinnamic acids were higher in the leaves than in the bilberry fruit. Hokkanen *et al.* [34] analyzed a methanolic extract of bilberry leaves by LC/TOF-MS and LC/MS-MS and identified thirty-five compounds. Other than the abundant chlorogenic acid (*trans*- and *cis*- form of 9) and its isomers, caffeoyl-shikimic acid (0.48% of the total combined area of all compounds), feroylquinic acid isomer (0.83%), and traces of caffeic acid (0.16%) were also quantified; percentages represent the relative share of each compound from the total combined peak area of all detected compounds in the leaves. The second significant group of phenolics is flavonoids. Quercetin-3-*O*-glucuronide is the most abundant flavonol and its concentration ranges from 70% to 93% of total flavonols [66]; other flavonols in bilberry leaves are quercetin-3-*O*-β-galactoside (4.06%), quercetin-3-*O*-(4’’-HMG)-α-rhamnoside (3.48%), quercetin-3-*O*-arabinoside (2.92%), quercetin-3-*O*-glucoside (0.99%), quercitrin (0.73%), and quercetin (0.03%), as well as three kaempferol glycosides (almost 1.5%) [19,34,66]. Hokkanen *et al.* [34] have in addition detected several other bioactive compounds in these leaves, such as flavan-3-ols comprising 2.0% of the total combined area of all compounds, six different isomers of cinchonain (18.5%), three proanthocyanidins (1.8%), and two coumaroyl iridoids (almost 1.0%). In another study, powdered leaves were extracted with diethyl ether and analyzed with regard to the triterpenoid content. Even though the triperpenes in the leaves comprised only the 4%–6% of those in the respective fruits, several compounds were identified in notable amounts (4.4–4.7 mg/g of dry leaf weight). The predominant compound was β-amyrin (23), followed by oleanane- and ursane-type triterpenes. The triterpene oleanolic and ursolic acids (22) were also identified [36].

The researchers have shown that the collection time of bilberry leaves greatly determines their phenolic content [66]. Hydroxycinnamic acid content strongly decreases during leaf development, while the content of flavonoids increases rapidly until mid-July, as flavonoids are formed later than phenolic acids in the biosynthetic process [38]. As the foliage ages, the color of bilberry leaves changes from green to red during early autumn; this alteration is attributed to differences in the phytochemical composition. Riihinen *et al.* [35] showed that red bilberry leaves contain anthocyanins, even though in very small concentration (0.882 mg/g frozen sample), in contrast with the green leaves where anthocyanins are absent. In addition, quercetin (14) (10.369 mg/g), kaempferol (15) (0.244 mg/g), *p*-coumaric (7) (6.007 mg/g), caffeic (1), or ferulic (4) acids (16.249 mg/g) are higher in the red bilberry leaves compared with the green ones (3.369, 0.171, 2.989, and 7.808 mg/g, respectively) [64]. On the other hand, both green and red leaves contain proanthocyanidins (red: 0.438 mg/g and green: 0.987 mg/g of frozen sample), especially procyanidin; thus, it has been suggested that these leaves should be viewed as a good source of proanthocyanidin-containing products and could be used in cosmetics and pharmaceuticals similarly to the phenolic compounds of green tea [35].

Bilberry has several traditional uses in folk medicine. Decoctions and infusions of its leaves are used for their diuretic, astringent, and antiseptic properties of the urinary tract. Bilberry leaf aqueous extracts are also useful as antibacterials and against inflammation, especially inflammation of the oral cavity [29]. In addition, a widespread use against diabetes has been reported [29].

Despite its regular and significant use as antidiabetic, *Vaccinium myrtillus* leaves have only been rarely investigated and the results are quite contradictory [39]. In alloxan-diabetic mice, a reduction in blood glucose levels by about 10% was reported in the early 1990s, but unfortunately, these experiments are not documented in detail [41]. Cignarella *et al.* [67] tested a dried hydroalcoholic extract of *V. myrtillus* leaves in streptozotocin-diabetic rats (3.0 g extract per kg body weight) and recorded lipid-lowering activity, *i.e.*, decrease by 39% of the triglycerides in the blood of dyslipidemic animals. In addition, they recorded a 26% decrease of plasma glucose levels, but the effect was characterized as “statistically, though not biologically significant”. Petlevski *et al.* [68] tested a multi-ingredient preparation composed of Myrtilli folium and nine other plant extracts, patented as an antidiabetic remedy in Croatia; they found a decrease in blood glucose and fructosamine levels in alloxan-induced non-obese diabetic mice. In studies where *Vaccinium* species were introduced in screening programs that aimed at identifying alpha-amylase inhibitors and activators of the human peroxisome proliferator-activated receptor gamma, bilberry leaf extracts showed some activity in both models [69] indicating possible antidiabetic properties. Finally, cinhonains might play significant role in the blood glucose lowering effect as they have been found to induce insulin secretion in both *in vitro* and *in vivo* experiments in rats [67].

Bilberry leaves have been investigated for their antistaphylococcal activity; significant effectiveness against *S. aureus* enhancing, at the same time, the bactericidal potential of vancomycin and linezolid in combination, which has been documented [32]. Finally, bilberry leaves have been explored for their protective activities against cancer. Flavonoids, caffeic acid, and chlorogenic acid were isolated from Sakhalin bilberry *Vaccinium smallii* leaves and were studied as cancer-preventive agents; they inhibited epidermal growth factor (EGF)-induced neoplastic transformation of mouse cells, without exerting any toxic effects [33].

## 6. Blueberry (*Vaccinium* sp.) Leaves

The term blueberries describes several different taxa of the genus *Vaccinium*; rabbiteye (*V. virgatum*), northern highbush (*V. corymbosum*), southern highbush (*V. formosum*), and lowbush (*V. angustifolium*) blueberries are the commonest.

Red dried leaves of *V. corymbosum* from Drama (region of Macedonia, Greece) were used for the preparation of a decoction (crude extract), which was further fractionated with the organic solvents ethyl acetate and butanol in our laboratory [70]. Analysis was performed by LC-ESI/MS and HPLC-DAD, and twenty different compounds were identified, mainly phenolic acids and flavonols. Interestingly, these two groups were in almost equal concentration in the crude extract (69.34 mg chlorogenic acid equivalents/g dry extract and 67.48 mg quercetin-3-*O*-galactoside equivalents/g dry extract, respectively) [70]; as in bilberry leaves, the most abundant compound was chlorogenic acid (9) (61.31 mg/g dry extract). LC-MS analysis showed the presence of quinic and caffeic acid (1), four myricetin glycosides, one kaempferol rutinoside, and seven quercetin glycosides, as well as quercetin aglycone (14). Hyperoside, isoquercetin, and rutin were the principle flavonoids (12.09 mg/g, 4.60 mg/g, and 3.16 mg/g dry extract). Moreover, we have detected proanthocyanidin B1/B2, kandelin and cinhonain. Kandelin was also reported in the *Vaccinium ashei* leaves [42], while cinhonains have been identified in bilberry leaves [34]. The absence of anthocyanins from the decoction was notable; however, it could also be attributed to the method of the extraction [70].

Wang *et al.* [40] published a study where 104 different cultivars of blueberries (rabbiteye, northern highbush, and southern highbush) were examined with respect to their phytochemical composition and antioxidant properties. Using HPLC–ESI–MS^2^ analysis, they identified three anthocyanins (cyanidin 3-*O*-glucoside, cyanidin 3-*O*-glucuronide, cyanidin 3-*O*- arabinoside) in the blueberry leaf methanolic extracts, even though in different quantities; northern highbush blueberries showed the higher total anthocyanin content (TAC). Nevertheless, TAC, which was measured semiquantitatively by linear regression of commercial standards, was almost ten times lower than that of the respective fruit in each cultivar, ranging from 0.09 to 4.4 mg cyanidin 3-*O*-glucoside equivalents/g dry weight. Leaf anthocyanins were not detected in some cultivars. Moreover, they detected four different proanthocyanidins but in very small amounts in the highbush blueberries (0.36–8.38 mg rutin equivalents/g dry weight) [40].

Leaf tissue maturation plays a significant role in the phytochemical composition of this species. Riihinen *et al.* [35] have showed that the red leaves of *V. corymbosum* contain higher amounts of quercetin (14) (3.530 mg/g frozen sample) and kaempferol (15) (0.505 mg/g), as well as of *p*-coumaric (7) (3.060 mg/g), caffeic (1) or ferulic (4) acids (19.870 mg/g) than the green leaves (1.784, 0.191, 0.490, 7.537 mg/g frozen sample, respectively). Solar radiation increases the content of the above-mentioned flavonols and hydroxycinnamic acids, probably due to their role in photo-protection [37]. This explains the higher content of those compounds in the red leaves compared to the green leaves. In addition, red leaves contain a very small amount of anthocyanins, which are absent from the green. On the other hand, prodelphinidins and procyanidins are present almost in the same quantity in both types of leaves (red: 0.485 mg/g, green: 0.468 mg/g frozen sample) [35].

Harris *et al.* [43] investigated the phytochemical profile of *V. angustifolium* leaves, demonstrating its high similarity with the highbush blueberry leaves. They identified ten different compounds in an ethanolic leaf extract. Chlorogenic acid (9) was the most abundant phenolic; it was 30 times more concentrated in the leaf extract (31.19 mg/g dry matter) than in the respective fruit (1.54 mg/g dry matter) and over 100 times more concentrated than in the respective stem or root extracts (0.09 and 0.03 mg/g dry matter, respectively). Moreover, they detected in significant amounts the flavan-3-ols epicatechin (20) and catechin (19) and in ratio roughly 1:1; they also quantified four quercetin glycosides (in total 9.65 mg/g dry matter), as well as quercetin aglycone (1.24 mg/g dry matter) (13). Quercetin-3-*O*-glucoside and quercetin-3-*O*-arabinoside accounted for 36% and 28%, respectively, of the quantified quercetin glycosides. Caffeic acid (1) was found in traces (0.36 mg/g dry matter); chlorogenic acid isomers (10, 11), quercetin-hexoside, quercetin-pentoside, and rutin were detected, but not quantified. Exactly the same compounds were quantified in another ethanolic extract of lowbush blueberry leaves, but in this case the measured quantities were almost three-fold higher than in the study of Harris *et al.* [44]. Anthocyanins were not detected in any of these studies.

Various members of the *Vaccinium* genus, other than bilberry, such as *Vaccinium macrocarpon* and *Vaccinium angustifolium*, are reputed to possess antidiabetic activity [71] and have been used extensively as traditional medicines for the treatment of diabetic symptoms [72]. Martineau *et al.* [44] demonstrated the significant antidiabetic activity of lowbush blueberry leaves *in vitro* with various cell-based bioassays. However, despite the widespread traditional use against diabetes, screening of current literature revealed the absence of investigations other than that of Martineau *et al.* [44].

The majority of studies focus on the antioxidant activities of blueberry leaves, which are related to their rich in phenolics composition [47]. In line with these findings, we have also demonstrated the high antioxidant capacity of *V. corymbosum* leaf decoction and its capability to bind iron ions [70]. In addition to ferrous chelation activity, in several *in vitro* experiments we have proven that quercetin (i) is totally oxidized by selenite ions, and (ii) it chelates calcium ions probably via the hydroxyl groups of A and B rings of the flavonoids. These observations were related to the protective activity that we have recorded against selenite-induced ocular cataract and selenite-induced oxidative damage in the brain and liver of neonatal rats [45,70]. Finally, highbush blueberry leaf extract acts as an antimicrobial agent, especially against *Salmonella typhymurium* and *Enterococcus faecalis* [46].

## 7. Cranberry (*Vaccinium* sp.) Leaves

Cranberries are a group of ecergreen dwarf shrubs or trailing vines in the subgenus of oxycoccus of the genus *Vaccinium*. In North America, cranberry may refer to *V. macrocarpon*, *V. microcarpon*, or *V. erythrocarpon*, whereas in Britain, cranberry usually refers to the native *V. oxycoccos*. In a recent comparative study, Teleszko *et al.* [73] analyzed the phytochemical composition of fruits and leaves of several berry species by UPLC-PDA/FL and LC/MS; among them, cranberry leaves were the second richest source of phenolics, richer than bilberry and blackcurrant leaves. The major polyphenolic group was proanthocyanidins, followed by flavonols. Proanthocyanidins (47.18 mg/g dry leaves), flavan-3-ols (27.76 mg/g), phenolic acids (2.36 mg/g), and flavonols (33.64 mg/g) were in higher concentration than in the respective fruits [73]. In addition, Neto *et al.* [74] have performed an HPLC-MS analysis of the phenolic profile of two cultivars, *i.e.*, in *Early Black* and *Howes*; the phenolic acids are mainly chlorogenic and neo-chlorogenic acid, as well as 3-*O*- and 5-*O*-coumaroylquinic acids. The principle flavonols were hyperoside and quercetin-3-*O*-rhamnoside, while quercetin-3-*O*-xyloside and quercetin-3-*O*-arabinoside were also detected. Procyanidin A2 was the identified catechin dimer. Finally, they documented two coumaroyl iridoid isomers (*trans*- and *cis*-form) previously reported in cranberry fruit [75]. All these compounds were in higher content in the cultivar *Early Black*.

The high phenolic content of cranberry leaves seems to be associated with the significant antioxidant potential that has been recorded with different methods [73]. Cranberries, however, are mostly known for their use against urinary tract infections. A randomized, double-blind, placebo-controlled cross-over experimental trial with 12 participants showed that the consumption of a cranberry-leaf beverage increased blood glutathione peroxidase activity, indicating its antioxidant capacity and inhibited the *ex vivo* adhesion of P-fimbriated *Escherichia coli* bacteria in urine, suggesting that cranberry leaf extracts may help to improve urinary tract health [76].

## 8. Lingonberry (*Vaccinium* *vitis*-*idaea*) Leaves

The green leaves of lingonberry (*Vaccinium vitis-idaea*) have similar phytochemical profile with those of bilberry [34,66]. Ieri *et al.* [66] and Hokkanen *et al.* [34] have quantified a great number of phenolics in the methanolic and hydro-alcoholic leaf extracts of lingonberry, respectively. In general, hydroxycinnamic acids and flavonols were the most abundant compounds. In the methanolic extract, flavonol content was higher than hydroxycinnamic acids, but in the hydro-alcoholic extract the opposite was observed, as expected. In both cases, the main acid was 2-*O*-caffeoylarbutin (12) (14%–35% of total phenols), which is not present in other berry leaves. Other phenolic acids detected in the methanolic extract were chlorogenic acid (3.55% of the total combined peak area of all compounds), coumaroyl quinic acid isomers (3.81%), caffeic acid (0.61%), *p*-coumaric acid (0.64%), and caffeoyl-shikimic acid (0.18%) [34]. However, lingonberry leaves contain coumaroyl- and caffeoyl-hexose hydroxyphenols (1.85% and 1.03% of the total combined peak area of all compounds) which are not present in bilberry leaves.

With respect to the flavonols, the principle compound was quercetin-3-*O*-(4’’-HMG)-α-rhamnoside in both studies comprising 5%–6% of total phenols in the hydroalcoholic extract and 32% of the total combined peak area of all compounds in the methanolic extract. Rutin (7.59% of the total combined peak area of all compounds), hyperoside (6.30%), and quercetrin (5.37%) were also detected in significant amounts in the methanolic extract, while traces of four more quercetin glycosides were also quantified. Furthermore, very small concentrations of kaempferol glycosides (8.03%), proanthocyanidins (1.4%), and coumaroyl iridoids (7.4%) were found [34].

Lingonberry leaves have similar traditional uses in folk medicine with the bilberry leaves [66]. They were mainly used as diuretics as well as for their antiseptic activity in urinary tract, probably due to the high content of tannins, especially arbutin and its derivatives [66]. Recently, the ethanolic extract of lingonberry leaves has shown significant antitussive, anti-inflammatory, and anti-catarrhal properties in rats [77]. In addition, Vyas *et al.* [78] has demonstrated that the acetone extract of these leaves possesses significant neuroprotective effect *in vitro* against glutamate-mediated excitotoxicity.

## 9. Conclusions

Despite their traditional uses, berry leaves are seldom used nowadays, in contrast to berry fruits, which are considered foods with significant health benefits. However, recent investigations have revealed that the traditional therapeutic properties of berry leaves may be valid. Moreover, the study of the phytochemical composition of berry leaves points out that they can be viewed as rich sources of bioactive natural products, e.g., tannins in raspberry and bilberry leaves, and chlorogenic acid in blueberry leaves, whereas other berry leaves, such as lingonberry, contain unique phenolics like arbutins. The phenolic compounds of the leaves are known antioxidant and anti-inflammatory agents (quercetin and kaempferol derivatives), and have hypoglycemic (cinchonains) and antimicrobial (ellagitannins) properties. Several studies and reviews have pointed out the anti-inflammatory activities of naturally-occurring compounds; the most effective are usually the aglycon forms of flavonoids (quercetin, kaempherol) and most of their actions are related to their ability to inhibit cytokine, chemokine release, and to be implicated in the molecular paths of the synthesis and/or action of adhesion molecules [79,80].

Epidemiological and meta-analyses studies suggest an inverse relationship between flavonoid-rich diets and development of many aging-associated diseases including cancers, cardiovascular disease, diabetes, osteoporosis, and neurodegenerative disorders [76]. Dietary flavonoids exert their anti-diabetic effects by targeting various cellular signaling pathways in pancreas, liver, and skeletal muscle; by influencing β-cell mass and function, as well as energy metabolism and insulin sensitivity in peripheral tissues [81]. Even though scientific literature specifically on the effectiveness of berry leaf consumption is extremely limited, the beneficial properties of individual flavonoids *in vitro* hold promise of positive outcomes. Nevertheless, *in vivo* studies with berry leaf extracts to evaluate modification of various biomarkers of disease and potential toxicity are needed. Additionally, bioavailability and pharmacokinetic studies in healthy human subjects, as well as carefully-designed and targeted intervention trials that would evaluate the impact of berry leaf-derived products on the prevention or the progress of specific disorders, are necessary. The forgotten berry leaves have just been “re-discovered” and may be viewed as sources of valuable bioactive compounds with health-promoting and disease-preventing properties.

## Figures and Tables

**Table 1:** An overview of the distribution of phenolic acids, flavonols, flavan-3-ols, ellagitannins (ET), proanthocyanidins (PACs), anthocyanins in blackcurrant (BC), blackberry (BB), raspberry (RP), bilberry (BI), highbush blueberry (H-BL), lowbush blueberry (L-BL), cranberry (CB) and lingonberry (LB) leaves.

	Compound Name	Berry Leaves							
**Phenolic Acids**	Chlorogenic acid	BC		RB	BI	H-BL	L-BL	CB	LGB
	Neo-chlorogenic acid	BC	BB			H-BL	L-BL	CB	
	Iso-chlorogenic acid	BC				H-BL			
	Caffeic acid	BC	BB	RB	BI	H-BL	L-BL		LGB
	Gallic acid	BC	BB						
	Ferulic acid	BC				H-BL			
	Quinic acid					H-BL			
	p-coumaric acid	BC	BB	RB		H-BL			LGB
	Coumaroyl-quinic acid						L-BL	CB	LGB
	Caffeoyl-shikimic acid/ Ferroyl-quinic acid isomer				BI				LGB
	Gentisic acid	BC							
	p-hydroxybenzoic acid/ vanillinic acid			RB					
	2-*O*-caffeoylarbutin								LGB
	Coumaroyl/caffeoyl-hexose hydroxyphenols								LGB
	Ellagic acid		BB	RB					
**Flavonols**	Quercetin		BB	RB	BI	H-BL	L-BL		
	Quercetin-3-*O*-rutinoside	BC		RB		H-BL			LGB
	Quercetin-3-*O*-galactoside	BC	BB	RB	BI	H-BL	L-BL	CB	LGB
	Quercetin-3-*O*-glucoside	BC		RB	BI	H-BL	L-BL		LGB
	Quercetin-3-*O*-glucuronide		BB	RB	BI				
	Quercetin-3-*O*-a-L-rhamnoside				BI	H-BL	L-BL	CB	LGB
	Quercetin-3-*O*-(4’’-HMG)-a-rhamnoside				BI				LGB
	Quercetin-3-*O*-arabinoside				BI	H-BL	L-BL	CB	LGB
	Quercetin-3-*O*-xyloside					H-BL		CB	LGB
	Quercetin-3-*O*-malonylglucoside	BC							
	Quercetin-3-*O*-glucosyl-6’’-acetate					H-BL			
	Quercetin-3-*O*-R-arabinofuranoside								LGB
	Kaempferol		BB			H-BL			
	Kaempferol-3-*O*-rutinoside	BC				H-BL	L-BL		
	Kaempferol-3-*O*-galactoside	BC							
	Kaempferol-3-*O*-glucoside	BC		RB					
	Kaempferol-3-*O*-glucuronide				BI	H-BL			
	Kaempferol-3-*O*-glucosyl-6’’-acetate	BC							
	Kaempferol-3-*O*-malonylglucoside	BC							
	kaempferol-(HMG)-rhamnoside								LGB
	Kaempferol-pentoside				BI				LGB
	Myricetin					H-BL			
	Myricetin-3-*O*-malonylglucoside	BC							
	Myricetin-3-*O*-rutinoside/-3- *O*-galactoside/-3-*O*-glucoside/-3-*O*-arabinoside/-3-*O*-xyloside					H-BL			
	Isorhamnetin-3-*O*-rutinoside/-3-*O*-glucoside	BC							
**Flavan-3-ols**	Catechin	BC	BB		BI		L-BL		LGB
	Epicatechin	BC	BB		BI		L-BL		
	Epigallocatechin/ Gallocatechin and its isomers	BC			BI				
	Epicatechin gallate methyl gallate		BB	RB					
**ETs**	Sanguiin H-6 /Lambertianin C		BB	RB					
	Lambertianin D			RB					
	Casuarinin		BB						
**PACs**	Cinchonains				BI		L-BL		
	Proanthocyanidin A1								LGB
	Proanthocyanidin A2							CB	LGB
	Proanthocyanidin B				BI				
	Kandelin A1/A2						L-BL		
	Procyanidins/Prodelphinidins						L-BL		
**Anthocyanins**	Delphinidin-3-*O*-glucoside/-3-*O*-rutinoside	BC							
	Cyanidin-3-*O*-glucoside	BC	BB			H-BL			
	Cyanidin-3-*O*-rutinoside	BC							
	Cyanidin-3-*O*-arabinoside/-3-*O*-glucuronide					H-BL			

BC: Blackcurrant, BB: Blackberry, RB: Rasberry, BI: Bilberry, H-BL: Highbush blueberry, L-BL: Lowbush blueberry, CB: Cranberry, LGB: Lingonberry, ETs: Ellagitannins, PACs: Proanthocyanidins

**Table 2:** Medicinal uses and biological properties of berry leaves.

	Blackcurrant		Red Raspberry		Blackberry	
**EMA**	*Traditional Medicinal Product* Minor articular pain Adjuvant in minor urinary complaints [13]		*Traditional Medicinal Product* Symptomatic relief of minor spasm associated with menstrual periods Symptomatic treatment of mild inflammation in the mouth or throat Symptomatic treatment of mild diarrhea [16]			
**Traditional uses**	Diaphoretic and diuretic agent Against diarrhea Against spasmodic cough Relief of rheumatic pain [14,15]		Labor stimulator [17] Relief of menstrual cramps Relief of diarrhea Astringent agent Anti-inflammatory agent (mouth, throat) Against chronic skin conditions Treatment of conjunctivitis [18]		Mouthwash against thrush, gum inflammations, mouth ulcers, sore throat Against respiratory problems Astringent agent Regulation of anemia, diarrhea, dysentery, cystitis, hemorrhoids [19]	
***In vitro*/*In vivo***	Antioxidant, Anti-inflammatory activity [14,20] Analgesic activity [15]		Antioxidant activity [21]		Antidiabetic/Hypoglycemic activity [22,23,24] Antimicrobial activity [25] Analgesic, Anti-inflammatory, Angiogenic activity [19,26,27]	
**Clinical trials**			Indications that it facilitates labor [28,29,30]			
	**Bilberry**	**Blueberry**		**Cranberry**		**Lingonberry**
**Traditional uses**	Diuretic, astringent and antiseptic agent for the urinary tract Antibacterial Anti-inflammatory Antidiabetic [31]	Antidiabetic agent [32,33]				Diuretic agent Antiseptic in urinary tract [31]
***In vitro*/*In vivo***	Antidiabetic activity [34,35,36] Anti-hyperlipidemic activity [37] Antistaphylococcal activity [38] Antioxidant, Anti-neoplastic activity [39]	Antioxidant, Anticataract [40] Neuroprotective activity [41] Antidiabetic activity [42] Antimicrobial activity [43]		Antioxidant activity [44]		Antitussive, Anti-inflammatory Anti-catarrhal activity [45] Neuroprotective activity [46]
**Clinical trials**				Antimicrobial agent—urinary tract protection Antioxidant activity [47]		

## Abbreviations

The following abbreviations are used in this manuscript:

DAD: Diode Array Detection
EGF: Epidermal Growth Factor
EMA: European Medicines Agency
ESI: Electronspray ionization
HMG: Hydroxymethylglutaric acid
HPLC: High Performance Liquid Chromatography
HPTLC: High Performance Thin Layer Chromatography
IR: Infrared spectroscopy
LC: Liquid Chromatography
MS: Mass Spectrometry
NMR: Nuclear Magnetic Resonance
TOF: Time-of-flight

## References

[1] European Commission (2010). Functional Foods.

[2] Beattie J., Crozier A., Duthie G.G. (2004). Potential health benefits of berries. Curr. Nutr. Food Sci..

[3] Mattila P., Hellstrom J., Torronen R. (2006). Phenolic acids in berries, fruits, and beverages. J. Agric. Food Chem..

[4] Maatta-Riihinen K.R., Kamal-Eldin A., Mattila P.H., Gonzalez-Paramas A.M., Torronen A.R. (2004). Distribution and contents of phenolic compounds in eighteen Scandinavian berry species. J. Agric. Food Chem..

[5] Maatta-Riihinen K.R., Kamal-Eldin A., Torronen A.R. (2004). Identification and quantification of phenolic compounds in berries of Fragaria and Rubus species (family Rosaceae). J. Agric. Food Chem..

[6] Kylli P. (2011). Berry Phenolics: Isolation, Analysis, Identification and Antioxidant Properties. Ph.D. Thesis.

[7] Häkkinen S., Heinonen M., Kärenlampi S., Mykkänen H., Ruuskanen J., Törrönen R. (1999). Screening of selected flavonoids and phenolic acids in 19 berries. Food Res. Int..

[8] Taruscio T.G., Barney D.L., Exon J. (2004). Content and profile of flavanoid and phenolic acid compounds in conjunction with the antioxidant capacity for a variety of northwest Vaccinium berries. J. Agric. Food Chem..

[9] Kahkonen M.P., Heinonen M. (2003). Antioxidant activity of anthocyanins and their aglycons. J. Agric. Food Chem..

[10] Koponen J.M., Happonen A.M., Mattila P.H., Torronen A.R. (2007). Contents of anthocyanins and ellagitannins in selected foods consumed in Finland. J. Agric. Food Chem..

[11] Gu L., Kelm M.A., Hammerstone J.F., Beecher G., Holden J., Haytowitz D., Gebhardt S., Prior R.L. (2004). Concentrations of proanthocyanidins in common foods and estimations of normal consumption. J. Nutr..

[12] Committee on Herbal Medicinal Products (HMPC) (2014). Call for Scientific Data for Use in HMPC Assessment Work on *Fragaria vesca* L., Folium.

[13] Committee on Herbal Medicinal Products (HMPC) (2012). Community Herbal Monograph on *Rubus idaeus* L., Folium.

[14] Committee on Herbal Medicinal Products (HMPC) (2009). Community Herbal Monograph on *Ribes nigrum* L., Folium.

[15] Committee on Herbal Medicinal Products (HMPC) (2009). Community Herbal Monograph on *Arctostaphylos uva-ursi* (L.) Spreng., Folium.

[16] Connolly T.J. (2003). Newberry crater: A ten-thousand-year record of human occupation and environmental change in the basin-plateau borderlands. Plains Anthropol..

[17] Riaz M.A., Rahman N.U. (2011). Antimicrobial screening of fruit, leaves, root and stem of *Rubus fruticosus* L.. J. Med. Plants Res..

[18] Sarkar A., Ganguly S.N. (1978). Rubitic acid, a new triterpene acid from *Rubus fruticosus*. Phytochemistry.

[19] Oszmianski J., Wojdylo A., Gorzelany J., Kapusta I. (2011). Identification and characterization of low molecular weight polyphenols in berry leaf extracts by HPLC-DAD and LC-ESI/MS. J. Agric. Food Chem..

[20] Raudsepp P., Kaldmae H., Kikas A., Libek A.V., Püssa T. (2010). Nutritional quality of berries and bioactive compounds in the leaves of black currant (*Ribes nigrum* L.) cultivars evaluated in Estonia. J. Berry Res..

[21] Committee on Herbal Medicinal Products (HMPC) (2012). Assessment Report on *Rubus idaeus* L., Folium.

[22] Oszmiański J., Wojdyło A., Nowicka P., Teleszko M., Cebulak T., Wolanin M. (2015). Determination of phenolic compounds and antioxidant activity in leaves from wild *Rubus* L. species. Molecules.

[23] Abu-Shandi K., Al-Rawashdeh A., Al-Mazaideh G., Abu-Nameh E., Al-Amro A., Al-Soufi H., Al-Ma’abreh A., Al-Dawdeyah A. (2015). A novel strategy for the identification of the medicinal natural products in *Rubus fruticosus* plant by using GC/MS technique: A study on leaves, stems and roots of the plant. Adv. Anal. Chem..

[24] Gudej J., Tomczyk M. (2004). Determination of flavonoids, tannins and ellagic acid in leaves from *Rubus* L. species. Arch. Pharm. Res..

[25] Zia-Ul-Haq M., Riaz M., de Feo V., Jaafar H., Moga M. (2014). *Rubus fruticosus* L.: Constituents, biological activities and health related uses. Molecules.

[26] Robinson G.M., Robinson R. (1931). A survey of anthocyanins. Biochem. J..

[27] Mukherjee M., Ghatak K.L., Ganguly S.N., Antoulas S. (1984). Rubinic acid, a triterpene acid from *Rubus fruticosus*. Phytochemistry.

[28] Greenway F.L., Zhijun L., Woltering E.A. (2007). Angiogenic Agents from Plant Extracts, Gallic Acid and Derivatives. U.S. Patent.

[29] Wang S.Y., Lin H.S. (2000). Antioxidant activity in fruits and leaves of blackberry, raspberry, and strawberry varies with cultivar and developmental stage. J. Agric. Food Chem..

[30] Scientific Committee of the British Herbal Medicine Association (1983). Rubus. British Herbal Pharmacopoeia.

[31] Jan G. (2003). Kaemferol and quercetin glycosides from *Rubus idaeus* L. leaves. Acta Polon. Pharm..

[32] Sadowska B., Paszkiewicz M., Podsedek A., Redzynia M., Rozalska B. (2014). *Vaccinium myrtillus* leaves and *Frangula alnus* bark derived extracts as potential antistaphylococcal agents. Acta Biochim. Pol..

[33] Mechikova G.Y., Kuzmich A.S., Ponomarenko L.P., Kalinovsky A.I., Stepanova T.A., Fedorov S.N., Stonik V.A. (2010). Cancer-preventive activities of secondary metabolites from leaves of the bilberry *Vaccinium smallii* A. Gray. Phytother. Res..

[34] Hokkanen J., Mattila S., Jaakola L., Pirttila A.M., Tolonen A. (2009). Identification of phenolic compounds from lingonberry (*Vaccinium vitis-idaea* L.), bilberry (*Vaccinium myrtillus* L.) and hybrid bilberry (*Vaccinium x intermedium* Ruthe L.) leaves. J. Agric. Food Chem..

[35] Riihinen K., Jaakola L., Karenlampi S., Hohtola A. (2008). Organ-specific distribution of phenolic compounds in bilberry (*Vaccinium myrtillus*) and “northblue” blueberry (*Vaccinium corymbosum*
*x V. angustifolium*). Food Chem..

[36] Szakiel A., Paczkowski C., Huttunen S. (2012). Triterpenoid content of berries and leaves of bilberry *Vaccinium myrtillus* from Finland and Poland. J. Agric. Food Chem..

[37] Jaakola L., Maatta-Riihinen K., Karenlampi S., Hohtola A. (2004). Activation of flavonoid biosynthesis by solar radiation in bilberry (*Vaccinium myrtillus* L.) leaves. Planta.

[38] Martz F., Jaakola L., Julkunen-Tiitto R., Stark S. (2010). Phenolic composition and antioxidant capacity of bilberry (*Vaccinium myrtillus*) leaves in Northern Europe following foliar development and along environmental gradients. J. Chem. Ecol..

[39] Helmstadter A., S chuster N. (2010). *Vaccinium myrtillus* as an antidiabetic medicinal plant—Research through the ages. Parmazie.

[40] Wang L.J., Wu J., Wang H.X., Li S.S., Zheng X.C., Du H., Xu Y.J., Wang L.S. (2015). Composition of phenolic compounds and antioxidant activity in the leaves of blueberry cultivars. J. Funct. Foods.

[41] Hänsel R., Keller K., Rimpler H., Schneider G. (1996). Drogen p–z. Hager’s Handbuch der Pharmazeutischen Praxis.

[42] Matsuo Y., Fujita Y., Ohnishi S., Tanaka T., Hirabaru H., Kai T., Sakaida H., Nishizono S., Kouno I. (2010). Chemical constituents of the leaves of rabbiteye blueberry (*Vaccinium ashei*) and characterisation of polymeric proanthocyanidins containing phenylpropanoid units and a-type linkages. Food Chem..

[43] Harris C.S., Burt A.J., Saleem A., Le P.M., Martineau L.C., Haddad P.S., Bennett S.A., Arnason J.T. (2007). A single HPLC-PAD-APCI/MS method for the quantitative comparison of phenolic compounds found in leaf, stem, root and fruit extracts of *Vaccinium angustifolium*. Phytochem. Anal..

[44] Martineau L.C., Couture A., Spoor D., Benhaddou-Andaloussi A., Harris C., Meddah B., Leduc C., Burt A., Vuong T., Mai Le P. (2006). Anti-diabetic properties of the Canadian lowbush blueberry *Vaccinium angustifolium* Ait. Phytomedicine.

[45] Ferlemi A.V., Makri O.E., Mermigki P.G., Lamari F.N., Georgakopoulos C.D. (2016). Quercetin glycosides and chlorogenic acid in highbush blueberry leaf decoction prevent cataractogenesis *in vivo* and *in vitro*: Investigation of the effect on calpains, antioxidant and metal chelating properties. Exp. Eye Res..

[46] Pervin M., Hasnat M.A., Lim B.O. (2013). Antibacterial and antioxidant activities of *Vaccinium corymbosum* L. leaf extract. Asian Pac. J. Trop. Dis..

[47] Piljac-Zegarac J., Belscak A., Piljac A. (2009). Antioxidant capacity and polyphenolic content of blueberry (*Vaccinium corymbosum* L.) leaf infusions. J. Med. Food.

[48] Pharmacopée Française (1996). Cassis (feuille de) Ribis nigri Folium.

[49] Trajkovski V. (1974). Resistance to *Sphaerotheca mors-uvae* (Schw.) Berk. in *Ribes nigrum* L.. Swed. J. Agric. Res..

[50] Vagiri M., Conner S., Stewart D., Andersson S.C., Verrall S., Johansson E., Rumpunen K. (2015). Phenolic compounds in blackcurrant (*Ribes nigrum* L.) leaves relative to leaf position and harvest date. Food Chem.

[51] Vagiri M., Ekholm A., Andersson S.C., Johansson E., Rumpunen K. (2012). An optimized method for analysis of phenolic compounds in buds, leaves, and fruits of black currant (*Ribes nigrum* L.). J. Agric. Food Chem..

[52] Tits M., Poukens P., Angenot L., Dierckxsens Y. (1992). Thin-layer chromatographic analysis of proanthocyanidins from *Ribes nigrum* leaves. J. Pharm. Biomed. Anal..

[53] Tits M., Angenot L., Poukens P., Warin R., Dierckxsens Y. (1992). Prodelphinidins from *Ribes nigrum*. Phytochemistry.

[54] Committee on Herbal Medicinal Products (HMPC) (2010). Assessment Report on Ribes nigrum L., Folium.

[55] Andersson J., Bosvik R., Von Sydow E. (1963). The composition of the essential oil of black currant leaves (*Ribes nigrum* L.). J. Sci. Food Agric..

[56] Tabart J., Franck T., Kevers C., Pincemail J., Serteyn D., Defraigne J.O., Dommes J. (2012). Antioxidant and anti-inflammatory activities of *Ribes nigrum* extracts. Food Chem..

[57] Grayer R.J., Kokubun T. (2001). Plant–fungal interactions: The search for phytoalexins and other antifungal compounds from higher plants. Phytochemistry.

[58] Xu Y., Zhang Y., Chen M. (2006). Effective Fractions of *Rubus fruticosus* Leaf, Its Pharmaceutical Composition and Uses for Prevention and Treatment of Diabetes. China Patent.

[59] Alonso R., Cadavid I., Calleja J.M. (1980). A preliminary study of hypoglycemic activity of *Rubus fruticosus*. Planta Med..

[60] Osório e Castro V.R. (2001). Chromium and zinc in a series of plants used in Portugal in the herbal treatment of non-insulinized diabetes. Acta Aliment..

[61] Durgo K., Belscak-Cvitanovic A., Stancic A., Franekic J., Komes D. (2012). The bioactive potential of red raspberry (*Rubus idaeus* L.) leaves in exhibiting cytotoxic and cytoprotective activity on human laryngeal carcinoma and colon adenocarcinoma. J. Med. Food.

[62] Brandely P. (2006). Raspberry leaf. British Herbal Compendium. A Handbook of Scientific Information on Widely Used Plant Drugs.

[63] Parsons M., Simpson M., Ponton T. (1999). Raspberry leaf and its effect on labour: Safety and efficacy. Aust. Coll. Midwives Inc. J..

[64] Jing Z., Pistilli M.J., Holloway A.C., Crankshaw D.J. (2010). The effects of commercial preparations of red raspberry leaf on the contractility of the rat’s uterus *in vitro*. Reprod. Sci..

[65] Simpson M. (2001). Raspberry leaf in pregnancy: Its safety and efficacy in labor. J. Midwifery Women's Health.

[66] Ieri F., Martini S., Innocenti M., Mulinacci N. (2013). Phenolic distribution in liquid preparations of *Vaccinium myrtillus* L. and *Vaccinium vitis idaea* L.. Phytochem. Anal..

[67] Cignarella A., Nastasi M., Cavalli E., Puglisi L. (1996). Novel lipid-lowering properties of *Vaccinium myrtillus* L. leaves, a traditional antidiabetic treatment, in several models of rat dyslipidaemia: A comparison with ciprofibrate. Thromb. Res..

[68] Petlevski R., Hadžija M., Slijepčević M., Juretić D. (2001). Effect of “antidiabetis” herbal preparation on serum glucose and fructosamine in NOD mice. J. Ethnopharmacol..

[69] Rau O., Wurglics M., Dingermann T., Abdel-Tawab M., Schubert-Zsilavecz M. (2006). Screening of herbal extracts for activation of the human peroxisome proliferator-activated receptor. Pharmazie.

[70] Ferlemi A.V., Mermigki P.G., Makri O.E., Anagnostopoulos D., Koulakiotis N.S., Margarity M., Tsarbopoulos A., Georgakopoulos C.D., Lamari F.N. (2015). Cerebral area differential redox response of neonatal rats to selenite-induced oxidative stress and to concurrent administration of highbush blueberry leaf polyphenols. Neurochem. Res..

[71] Chambers B.K., Camire M.E. (2003). Can cranberry supplementation benefit adults with type 2 diabetes?. Diabetes Care.

[72] Jellin J.M., Gregory P.J., Batz F., Hitchens K. (2005). Natural medicines comprehensive database. Pharm. Lett./Prescr. Lett..

[73] Teleszko M., Wojdyło A. (2015). Comparison of phenolic compounds and antioxidant potential between selected edible fruits and their leaves. J. Funct. Foods.

[74] Neto C.C., Salvas M.R., Autio W.R., van den Heuvel J.E. (2010). Variation in concentration of phenolic acid derivatives and quercetin glycosides in foliage of cranberry that may play a role in pest deterrence. J. Am. Soc. Hortic. Sci..

[75] Turner A., Chen S.N., Nikolic D., van Breemen R., Farnsworth N.R., Pauli G.F. (2007). Coumaroyl iridoids and a depside from cranberry (*Vaccinium macrocarpon*). J. Nat. Prod..

[76] Mathison B.D., Kimble L.L., Kaspar K.L., Khoo C., Chew B.P. (2014). Consumption of cranberry beverage improved endogenous antioxidant status and protected against bacteria adhesion in healthy humans: A randomized controlled trial. Nutr. Res..

[77] Wang X., Sun H., Fan Y., Li L., Makino T., Kano Y. (2005). Analysis and bioactive evaluation of the compounds absorbed into blood after oral administration of the extracts of *Vaccinium* vitis-idaea in rat. Biol. Pharm. Bull..

[78] Vyas P., Kalidindi S., Chibrikova L., Igamberdiev A.U., Weber J.T. (2013). Chemical analysis and effect of blueberry and lingonberry fruits and leaves against glutamate-mediated excitotoxicity. J. Agric. Food Chem..

[79] Hämäläinen M., Nieminen R., Vuorela P., Heinonen M., Moilanen E. (2007). Anti-inflammatory effects of flavonoids: Genistein, kaempferol, quercetin, and daidzein inhibit STAT-1 and NF-κB activations, whereas flavone, isorhamnetin, naringenin, and pelargonidin inhibit only NF-κB activation along with their inhibitory effect on iNOS expression and NO production in activated macrophages. Med. Inflamm..

[80] Calixto J.B., Campos M.M., Otuki M.F., Santos A.R. (2004). Anti-inflammatory compounds of plant origin. Part II. Modulation of pro-inflammatory cytokines, chemokines and adhesion molecules. Planta Med..

[81] Babu P.V., Liu D., Gilbert E.R. (2013). Recent advances in understanding the anti-diabetic actions of dietary flavonoids. J. Nutr. Biochem..

